# A collaborative network trial to evaluate the effectiveness of implementation strategies to maximize adoption of a school-based healthy lunchbox program: a study protocol

**DOI:** 10.3389/fpubh.2024.1367017

**Published:** 2024-03-27

**Authors:** Courtney Barnes, Jannah Jones, Luke Wolfenden, Katie Robertson, Anna Lene Seidler, Jennifer Norman, Pip Budgen, Megan Mattingly, Carla Piliskic, Lisa Moorhouse, Jennifer Mozina, Jennifer Plaskett, Sarah McDermott, Sara Darney, Cecilia Vuong, Nina Douglass, Kara McDonnell, Rachel Sutherland

**Affiliations:** ^1^Hunter New England Population Health, Hunter New England Local Health District, Wallsend, NSW, Australia; ^2^School of Medicine and Public Health, University of Newcastle, Callaghan, NSW, Australia; ^3^Population Health Research Program, Hunter Medical Research Institute, New Lambton Heights, NSW, Australia; ^4^National Centre of Implementation Science, University of Newcastle, Callaghan, NSW, Australia; ^5^NHMRC Clinical Trials Centre, University of Sydney, Sydney, NSW, Australia; ^6^Health Promotion Service, Illawarra Shoalhaven Local Health District, Warrawong, NSW, Australia; ^7^School of Health and Society, Faculty of the Arts, Social Sciences and Humanities, University of Wollongong, Wollongong, NSW, Australia; ^8^Health Promotion, Murrumbidgee Local Health District, Albury, NSW, Australia; ^9^Health Promotion Unit, Population Health, Nepean Blue Mountains Local Health District, Penrith, NSW, Australia; ^10^Health Equity, Promotion and Prevention Service, South Eastern Sydney Local Health District, Darlinghurst, NSW, Australia; ^11^Population Health, Southern NSW Local Health District, Queanbeyan, NSW, Australia; ^12^Centre for Population Health, Western Sydney Local Health District, North Parramatta, NSW, Australia; ^13^Western NSW Health Promotion, Western NSW Local Health District, Dubbo, NSW, Australia; ^14^Health Promotion Service, South Western Sydney Local Health District, Liverpool, NSW, Australia; ^15^Health Promotion Service, Central Coast Local Health District, Gosford, NSW, Australia; ^16^Population Health Promotion, Northern Sydney Local Health District, Brookvale, NSW, Australia

**Keywords:** public health nutrition, children, school, randomized controlled trial, Master Protocol

## Abstract

**Introduction:**

An important impediment to the large-scale adoption of evidence-based school nutrition interventions is the lack of evidence on effective strategies to implement them. This paper describes the protocol for a “Collaborative Network Trial” to support the simultaneous testing of different strategies undertaken by New South Wales Local Health Districts to facilitate the adoption of an effective school-based healthy lunchbox program (‘SWAP IT’). The primary objective of this study is to assess the effectiveness of different implementation strategies to increase school adoption of the SWAP across New South Wales Local Health Districts.

**Methods:**

Within a Master Protocol framework, a collaborative network trial will be undertaken. Independent randomized controlled trials to test implementation strategies to increase school adoption of SWAP IT within primary schools in 10 different New South Wales Local Health Districts will occur. Schools will be randomly allocated to either the intervention or control condition. Schools allocated to the intervention group will receive a combination of implementation strategies. Across the 10 participating Local Health Districts, six broad strategies were developed and combinations of these strategies will be executed over a 6 month period. In six districts an active comparison group (containing one or more implementation strategies) was selected. The primary outcome of the trial will be adoption of SWAP IT, assessed via electronic registration records captured automatically following online school registration to the program. The primary outcome will be assessed using logistic regression analyses for each trial. Individual participant data component network meta-analysis, under a Bayesian framework, will be used to explore strategy-covariate interactions; to model additive main effects (separate effects for each component of an implementation strategy); two way interactions (synergistic/antagonistic effects of components), and full interactions.

**Discussion:**

The study will provide rigorous evidence of the effects of a variety of implementation strategies, employed in different contexts, on the adoption of a school-based healthy lunchbox program at scale. Importantly, it will also provide evidence as to whether health service-centered, collaborative research models can rapidly generate new knowledge and yield health service improvements.

**Clinical trial registration:**

This trial is registered prospectively with the Australian New Zealand Clinical Trials Registry (ACTRN12623000558628).

## Introduction

Dietary risk factors are a leading cause of preventable death and disability (1). Reducing dietary risks is recommended to improve child health and mitigate future burdens of chronic disease (2). In Australia, for example, 96% of children do not consume sufficient serves of vegetables, while discretionary foods (i.e., foods high in added sugar, saturated fat and sodium) account for over one-third of children’s daily energy intake (3). Schools provide universal access to children aged over 5 years, and are a setting recommended for nutrition interventions in chronic disease prevention internationally (4–6). In countries such as Australia, food brought to school (from home) packed in school ‘lunchboxes’ are used daily by 90% of students, (7) and contribute up to 30–50% of a child’s daily energy intake (7). As approximately 40% of foods in lunchboxes are discretionary (8) improving the packing of healthy foods for child consumption at school provides a considerable opportunity for chronic disease prevention.

Systematic reviews suggest that school-based healthy lunchbox interventions can improve student nutritional intake (9). In Australia, a series of randomized controlled trials of a healthy lunchbox program, known as ‘SWAP IT’ were recently conducted in 34 primary schools with 4,600 children (10, 11). The program supports parents and carers to make simple ‘swaps’ aligned to dietary guidelines, (12) replacing discretionary food and beverage items with comparable core (nutrient dense) items. It is comprised of three broad program components: (i) school food (lunchbox) guidelines; (ii) messages and hard copy resources to parents and carers; and (iii) curricula resources for teachers. Across these randomized trials, the program was found to significantly improve child diet quality, energy intake and weight status, and was acceptable to both parents and teachers (10, 11). A subsequent comparative effectiveness randomized trial found no difference in effectiveness between the messages and parent booklets combined, compared with those two components plus school-based curriculum and policy resources on student dietary outcomes.

Given the reported benefits of SWAP IT on child health, (10, 11) broad implementation in schools has the potential to make a significant contribution to improving public health nutrition. An important impediment to the large scale adoption of effective school nutrition initiatives, however, is a lack of published evidence of effective strategies to implement them (13). A recent Cochrane review of implementation strategies for school-based health promotion programs identified few randomized controlled trials of strategies to implement policies and practices promoting healthy eating, particularly ‘at scale’ (defined by the authors as 50 or more schools) (13). Furthermore, strategies identified as effective in improving implementation in one jurisdiction (e.g., Local Health District), may not be effective, appropriate or feasible for application in another. Similarly, differing capacities (e.g., resources or infrastructure) of agencies responsible for undertaking or supporting program implementation may mean an effective implementation strategy in one jurisdiction may not be feasible to execute in another. Such issues must be addressed if effective interventions are to be adopted at a population level (at scale).

As in clinical services, systematic reviews and best practice guidelines identify evidence-based programs and practices that can be employed in community settings to reduce child dietary risks. As such, within devolved health systems such as Australia, different health services will often seek to address the same disease risk or health condition, using the same intervention (e.g., guideline concordant care) at the same time (14). These services, however, operate in different contexts, with different capacities and resource constraints. As a result, there is often natural heterogeneity in the strategies that health services employ to support the implementation of programs in schools and other clinical and community settings to improve dietary (and other) outcomes. This convergence of objective (to implement a similar intervention), but heterogeneity in context and strategies used to implement school-based programs, presents an attractive opportunity to learn about the types of implementation strategies that may be effective in different contexts. Specifically, the coordinated evaluation of implementation efforts across a network of health services, and the establishment of processes to share and learn from the findings, may provide a mechanism for rapid evidence generation, and health system improvement ‘at scale’. Such collaborative and data-driven models of working are also consistent with recommendations for the development of ‘learning health system’ approaches to healthcare improvement (15).

Broadly, Master Protocols represent an approach that could be used to facilitate coordinated and collaborative research, learning and improvement (16). Master Protocols refer to designs employing coordinated approaches to assess the effects of interventions within a unifying overall trial structure (16). This infrastructure, including a centralized trial protocol and governance, facilitates the standardization of study processes and procedures, including recruitment, evaluation and data collection, analysis, and reporting (17). Although frequently used to test pharmacological interventions, (18–20) this type of trial design is not broadly used within community-based interventions and to our knowledge, has not previously been used to assess the effectiveness of strategies on school implementation of health promotion programs. Employing this type of trial design would be a novel transformation from how health promotion programs, and strategies to support their implementation, are conventionally tested. Currently, few trials test the effectiveness of strategies to improve the implementation of such programs, (21) and those that do often employ different research designs and measures. This impedes cross-study synthesis, and also fails to address the issue of context, with strategies that effectively improve implementation in one context potentially unsuitable or ineffective in another (22). Comparatively, Master Protocol designs allow for the examination of multiple hypotheses, (23) such as the effects of a variety of implementation or scale-up strategies on school implementation of health promotion programs, or differences in effectiveness for different population groups.

Following demonstration of the effectiveness and acceptability of the SWAP IT program, (10, 11) three Local Health Districts (LHDs) from across New South Wales (NSW), Australia, expressed interest in supporting the implementation of this program in their LHD. In this context, and drawing on research design principles of Master Protocols and prospective meta-analysis methodology, (24) a pilot collaboration was formed that networked three LHDs and the University of Newcastle (National Centre of Implementation Science) (25) to undertake a harmonized evaluation of strategies used within each LHD to support the adoption of the SWAP IT program, and to share learning from these evaluations across participating LHDs (26). The collaboration was supported by shared implementation strategy development processes, governance structures, centralized data collection infrastructure, and a community of practice (26). While collaboration across and flexibility within LHDs for the implementation of various health promotion programs has occurred routinely among NSW LHDs over time, a formal evaluation of such a collaborative approach had not been undertaken. The pilot found the collaborative model was highly acceptable to all parties, (26) and strategies employed yielded significant, but contextually dependent improvements in program adoption.

Based on these encouraging findings, the collaborative approach is now being employed across 10 of the 15 LHDs (67%) in NSW. This paper describes the protocol for what we term a “Collaborative Network Trial” to support the simultaneous testing of different implementation strategies undertaken by 10 LHDs in NSW, Australia to facilitate the adoption of the SWAP IT program at scale.

## Objectives

As such, the primary objective of this study is to assess, using individual level participant (in this case ‘school’) data (IPD), the effectiveness of different implementation strategies employed by 10 NSW LHDs to increase school adoption of the SWAP IT program. Secondary objectives of the study are to: (1) explore the effects of different implementation strategy components and contextual factors on the school-level adoption of SWAP IT using pooled individual level data across all trials; (2) assess the acceptability of the implementation strategies to school principals; and (3) assess the sustainability of SWAP IT within schools that adopted the program at 18-months.

## Materials and methods

### Context

LHDs are NSW Government funded health services responsible for providing or supporting the provision of health promotion services to address the leading risk factors for chronic disease in their community. The NSW Ministry of Health provides funding to LHDs to support the implementation of state-wide health promotion programs (27). These health promotion programs are often developed by employing a multi-sectoral approach, involving health (e.g., LHD health promotion practitioners), policy (e.g., NSW Ministry of Health) and education stakeholders (e.g., Department of Education) to maximize the alignment of the programs with the priorities of the school sector, such as fit of the program with the school curriculum and student wellbeing policies. All NSW LHDs have received funding to facilitate the implementation of healthy eating and physical activity policies and practices in NSW primary schools for over a decade as part of the NSW Healthy Children’s Initiative (27). This involves LHD health promotion staff engaging with all schools in their region to deliver training, education and other health promotion activities to support schools to implement healthy eating and physical activity policies and practices. Although healthy lunchboxes have historically been a focus for health promotion activities in some LHDs and non-government organisations (e.g., Cancer Council NSW), the funding provided by NSW Ministry of Health did not explicitly focus on a formal school-based program to support the packing of healthy lunchboxes. In addition, while a core component of health promotion practice, Health Promotion Unit capability to undertake research and evaluation of health promotion activity has been found to vary across LHDs (28).

### Ethics and trial registration

The research will be conducted and reported in accordance with the requirements of the Consolidated Standards of Reporting Trials (CONSORT) Statement (29). Ethics approval has been obtained via the following Human Research Ethics Committees: Hunter New England (2019/ETH12353); University of Newcastle (09/07/26/4.04); NSW of Department of Education (2018247); and the Maitland-Newcastle, Sydney, Wollongong, Bathurst, Parramatta, Wagga Wagga and Canberra-Goulburn Catholic Dioceses. This trial is also registered prospectively with the Australian New Zealand Clinical Trials Registry (ACTRN12623000558628). The protocol is reported according to the Standard Protocol Items: Recommendations for Interventional Trials (SPIRIT) (Supplementary Files 1, 2) (30).

### Study design and setting

Within a Master Protocol framework, (16) we will undertake a Collaborative Network Trial. Specifically, independent randomized controlled trials to test strategies to implement or improve health care occurring at different sites (LHDs) will be undertaken by the Health Promotion Units at each LHD. The key trial methods, measures and data collection processes will be harmonized with agreement across sites to provide individual school-level data for planned pooled analyses as part of a collaborative, following a prospective meta-analysis framework (24). The design allows for heterogeneity or natural variation in the implementation strategies being tested and the contexts (i.e., sites) they are tested in (16). The study builds on a pilot network trial to implement the scale-up of SWAP IT program in three LHDs (13).

### Sample and participants

The study will be conducted with primary and combined schools located across 10 LHDs in NSW, Australia. The state of NSW is socioeconomically and geographically diverse (31). Department of Education (DoE), Catholic Schools NSW and Association of Independent Schools of NSW primary and combined schools located within the LHDs of Murrumbidgee, Hunter New England, Sydney, Western Sydney, South Western Sydney, South Eastern Sydney, Northern Sydney, Western NSW, Nepean Blue Mountains, and Illawarra Shoalhaven will be included in the study. These LHDs have partnered with the research team to participate in a separate trial occurring concurrently with other primary and combined schools in their region (ACTRN12623000145606). As such, LHD staff are well engaged in the research and infrastructure and resources to support the research (e.g., regular meetings with research sites/LHDs, data collection systems and staff) are in place.

A list of potentially eligible schools located within NSW will be sourced from a publicly accessible database (*n* = 3,183) (32). The research team will apply the following criteria prior to commencing the study to identify schools that are eligible for inclusion. Primary and combined schools located within the participating LHDs who cater for at least one primary school year and have not implemented the SWAP IT program will be eligible to participate. Only schools that do not use the Audiri parent communication app will be eligible, as these schools are participating in another trial being conducted concurrently by the research team (ACTRN12623000145606). The following schools will be excluded from the study sample: schools with special purposes (e.g., schools catering exclusively for children requiring specialist care, hospital schools, distance education schools and environmental education centers) (*n* = 206); schools with secondary students only (*n* = 544); schools identified as early learning centers (*n* = 6); schools located outside of the partnering LHDs (*n* = 483); schools who have already implemented SWAP IT (*n* = 208); and schools that have previously participated or currently participating in separate SWAP IT trials (*n* = 394). The total sample of eligible schools is 1,342.

All schools that meet the eligibility criteria outlined above will be included in the study as part of usual service delivery provided by LHD health promotion staff to support schools to implement a range of healthy eating and physical activity policies and practices. Eligible schools will be invited to participate in the secondary data collection component of the study, specifically the follow-up survey conducted with school principals (described below). Schools will be recruited for the follow-up data collection via an invitation email containing a link to an online survey and a study information statement outlining the purpose of the research and their involvement. Schools that are yet to complete the survey will receive up to three reminder prompts via telephone or email by the research team to encourage completion. Recruitment for the data collection component commenced in November 2023 and concluded in December 2023.

### Randomization and blinding

Prior to the delivery of the first scale-up strategy, schools within each LHD will be randomly allocated to either the intervention or control condition using a computerized random number function in a 1:1 (intervention: control) ratio. Randomization will be stratified by school size and social socio-economic location, as determined by Socio-Economic Indexes for Areas categorization using school postcodes, (33) given the socio-economic association with implementation of school nutrition programs (34). Randomization will be completed by a statistician not otherwise involved in the trial. Due to the nature of the intervention, participants will not be blinded to group allocation. However, research staff assessing the outcomes at follow-up will be blinded.

### Implementation strategies

A series of implementation strategies were developed with the aim of maximizing school adoption of the SWAP IT program in eligible schools that have not yet adopted the program. These implementation strategies were developed for each of the LHDs independently, based on their existing capacities and local contexts. Implementation strategies for each participating LHD (‘site’) were co-designed by LHD health promotion staff and other stakeholders, with support provided by National Centre of Implementation Science (NCOIS) implementation scientists and SWAP IT developers from the University of Newcastle. The development process included: (i) planning workshops facilitated by University staff that drew on tacit knowledge and experience of health promotion staff who had considerable experience working with schools; (ii) evidence regarding barriers to school adoption and implementation of SWAP IT collected by the research team as part of previous SWAP IT trials, (iii) data from systematic reviews and pilot trials regarding the effectiveness of strategies to facilitate adoption (32). During the workshops, theoretical framework tools were used to facilitate the selection of strategies to address barriers that were aligned to individual LHD capacity and contexts (35–37). Processes may have also been undertaken by LHDs to identify strategies to support access and engagement of priority populations within their region to ensure school adoption and implementation of SWAP IT does not further exacerbate health inequities. This may have included consultation and engagement processes with Aboriginal, or Culturally and Linguistically Diverse individuals, groups or stakeholders.

Across the 10 participating LHDs, six broad implementation strategies to maximize school adoption of SWAP IT emerged. The combination of these six strategies employed by each LHD will differ and will be executed over a period of 6 months. Once a school adopts SWAP IT, they will not receive any subsequent implementation strategies, and will select which school term they would prefer to receive the program. The SWAP IT messages are delivered weekly to parents and carers via usual school-parent communication channels for one school term (one message per week), followed by two messages per term on an ongoing basis. A school must adopt SWAP IT in order to receive the program.

The implementation strategies executed by each LHD are described below and in Table 1, with the timeline for the delivery of the implementation strategies outlined in Table 2.

**Table 1:** Implementation strategies delivered by each Local Health District.

Local Health District	Group	Sector support and endorsement	Local facilitation	Educational meetings	Educational materials	Local opinion leaders	Audit and feedback
LHD 1	Intervention						
	Control						

LHD 2	Intervention						
	Control						

LHD 3	Intervention						
	Control						

LHD 4	Intervention						
	Control						

LHD 5	Intervention						
	Control						

LHD 6	Intervention						
	Control						

LHD 7	Intervention						
	Control						

LHD 8	Intervention						
	Control						

LHD 9	Intervention						
	Control						

LHD 10	Intervention						
	Control						

**Table 2:** Timeline for the delivery of the implementation strategies.

School Term	TERM 2 2023											SCHOOL HOLIDAY		TERM 3 2023										SCHOOL HOLIDAY		TERM 4 2023			
Term week	1		2	3	4	5	6	7	8	9	10	1	2	1	2	3	4	5	6	7	8	9	10	1	2	1	2	3	4
Strategy	Local-level Health endorsement letter	Printed information pack				Webinar	Tailored contact one							Tailored contact two							Admin manager email	Parent committee pack	Email		State-level Health endorsement letter				

#### Sector support and endorsement

Policy makers from Health will target principals to communicate, support and endorse the program and its outcomes, its alignment to sector policies and recommend its adoption. This endorsement will occur via a maximum of two targeted letters or emails developed by the research team, approved and endorsed by local and state-level Health partners. The letters or emails will also contain a link to resources and the enrolment website. As an additional strategy, some LHDs (outlined in Table 1) will use their existing connections to obtain endorsement for the program from local educational and wellbeing liaisons within the NSW Department of Education. This endorsement will be promoted to schools via an email distributed by the liaisons directly to schools receiving this strategy.

#### Local facilitation

Health promotion staff from LHDs have developed strong and trusted local relationships with schools for over a decade and represent credible sources of local nutrition expertise. LHD health promotion staff will use up to two of their existing planned school contacts, conducted via telephone call or face-to-face meeting, to assess interest in the SWAP IT program, address any school-specific barriers to adoption, and facilitate goal setting and action planning. Scripts developed by the research team to guide the local facilitation will incorporate motivational interviewing techniques to be employed by health promotion staff to address school barriers to program adoption.

#### Develop and distribute educational materials

Targeted at principals to address perceived barriers to adoption, the strategy will initially aim to create tension for change (e.g., via outlining parent and carer interest and expectations); and then communicate the attractive program attributes (e.g., simplicity, no-cost). This communication will consist of up to two contacts, including a printed information pack (consisting of a flyer, SWAP IT pen and example parent booklet) at the commencement of the intervention period followed by an email to promote the program. As an additional strategy, one LHD will offer printed parent booklets promoting the SWAP IT program to all parents and carers with children commencing the following school year within their school kindergarten orientation packs along with a flyer encouraging the school principal or wellbeing coordinator to adopt the program.

#### Local opinion leaders

Promotional materials, including one printed information pack (consisting of a flyer and example SWAP IT parent booklet) and one email, will be delivered to other leaders that may be influential in a schools decision to adopt health promotion programs, specifically the school administration manager and parent committee. The aim of these materials is to promote the SWAP IT program and encourage school adoption.

#### Audit and feedback

Data and feedback on school adoption of SWAP IT will be automatically captured through electronic registration records and be provided to schools via other implementation strategies, including educational materials, local facilitation and local opinion leaders. For example, educational materials provided to principals, school administration managers and parent committees will include information on the number of schools that have registered for SWAP IT, a link to view an online list of schools have already adopted the program (to create tension for change and social norms) and provide instruction on how the school can also register for the program.

#### Educational meeting

Health promotion staff from LHDs will conduct one webinar with schools within their LHD to assess interest in the SWAP IT program and address any barriers to adoption. Webinar content will be developed by the research team in collaboration with health promotion staff.

### Control group and contamination

Registration for the SWAP IT program is publicly available and freely accessible for all schools, including schools allocated to the control group. The implementation strategies to be delivered to the control group across LHDs is described in Table 1. For most schools allocated to the control group, the comparison will be ‘no implementation support’ or a singular strategy. Execution of the implementation strategies will be monitored centrally by the research team in consultation with health promotion staff from each LHD to minimize risk of contamination. Nonetheless, school exposure to the implementation strategies will be assessed at follow-up via an online or telephone survey with school principals (described below).

### Study outcomes and data collection

Trial outcomes were discussed and agreed upon by participating LHDs (Table 3). Data collection for all trial outcomes were harmonized across all LHDs and will be collected centrally by the research team at the University of Newcastle. The centralisation of data collection represented an efficient means of collecting and managing data for all participating LHDs. All demographic, operational and trial outcome measures are harmonized (i.e., identical item, measure and data collection method) to facilitate comparability and analysis. Each participating LHD will retain access to their trial dataset.

**Table 3:** Study outcomes and sample assessed.

Outcome	Timepoint	Study sample assessed
Adoption of the SWAP IT program	Baseline and 9 months post baseline	All schools within the study sample
Acceptability of the implementation strategies	9 months post baseline	Sub-sample of 243 schools
Implementation of the SWAP IT program	9 months post baseline	Schools within the sub-sample of 243 that have adopted SWAP IT
Sustainability of the SWAP IT program	18 months post baseline	All schools within the study sample that have adopted SWAP IT
School characteristics	Baseline	All schools within the study sample

#### Primary outcome

Adoption of the SWAP IT program, defined as the number of schools who register for the lunchbox nutrition program (SWAP IT), will be assessed within schools allocated to the intervention and control group via electronic registration records captured automatically following school registration to SWAP IT. No additional data collection is required to assess the primary outcome. As part of the registration process, schools provide consent for the de-identified registration data to be used for research and evaluation purposes. This outcome will be assessed at baseline and approximately 9 months after baseline data collection.

#### Secondary outcomes

Acceptability of implementation strategies, defined as the perception among principals that the implementation strategies are agreeable, palatable or satisfactory, will be assessed in a telephone or online survey with school principals at 9-month follow-up. School principals will be asked if they recall receiving each of the implementation strategies during the intervention period. For strategies the participants recall receiving, they will be asked to rate how acceptable they found the strategy on a 5-point Likert scale (1 = not acceptable; 5 = very acceptable) (38). Principals from 243 Catholic and Independent primary schools located across five LHDs (LHD 1; LHD 5; LHD 7; LHD 8; LHD 9) will be invited to participate in the survey. These LHDs have been selected as they are employing diverse combinations of the implementation strategies (Table 1). Including schools from these LHDs in the survey will ensure the acceptability of all employed strategies (across the 10 LHDs) will be assessed and ensures the data collection remains feasible to be conducted within the study timeline.

Implementation of the SWAP IT program, defined as the extent to which the SWAP IT program components were delivered by the school to parents, will be assessed in the telephone or online survey (described above) with a sub-sample of 243 school principals at 9-months follow-up. Schools will be asked to report if they implemented the SWAP IT program at their school, and what program components were implemented (i.e., parent messages; school lunchbox guidelines; curriculum resources; parent and carer resources).

Sustainability of the SWAP IT program, defined as continued school use of the lunchbox nutrition program (SWAP IT) at 18 months after baseline data collection, will be assessed via electronic registration records captured automatically following school registration to SWAP IT.

School characteristics, including postcode, total student enrolments, geographic location (urban, regional, rural and remote), proportion of Aboriginal student enrolments, and proportion of students that speak a language other than English at home, were obtained from a publically accessible Australian Curriculum, Assessment and Reporting Authority (ACARA) database (39).

### Sample size and data analysis

We are anticipating a sample of at least 30 schools per group (and an average of 60 per group) in trials of each of the 10 participating LHDs. Descriptive statistics, including proportions, means and standard deviations, will be used to describe school characteristics, adoption, implementation and sustainability of SWAP IT, as well as the acceptability of the implementation strategies.

Analyses of trial outcomes will be undertaken under an intention to treat framework separately for each trial. For assessment of school level program adoption, the primary trial outcome, between-group differences, will be assessed using logistic regression. The model will include a term for treatment group (intervention vs. control) and pre-specified covariates prognostic of the outcome. Little, if any, missing primary outcome data is anticipated at follow-up, as program adoption is recorded automatically for all participating schools. Nonetheless, we will employ multiple imputation for any missing data in the event that schools withdraw from the study and request that their data are not used. All statistical tests will be 2 tailed with alpha of 0.05. Assuming adoption of the program by 10% in the comparison group, a sample size of approximately 30 schools per group will be sufficient to detect an absolute difference between groups of 30%, with 80% power and an alpha of 0.05.

We will employ component IPD component network meta-analysis to compare and rank the effects from all the tested strategies on the primary trial outcome (40). For this analysis we will also include the three randomized controlled trials from the pilot, (26) expanding the network and providing pooled individual level data from 13 randomized controlled trials. We will explore combining ‘educational meetings and educational materials’ into a single component for analysis given their shared underlying behavioral targets. We will adjust for prognostic factors and exploration of strategy—covariate interactions to identify if and to what extent effects vary by participant, population or other contextual factors (effect modifier) (40). We will also employ component network meta-analyses to model additive main effects (separate effects for each element or component of an implementation strategy); two way interactions (synergistic/antagonistic effects of components), and full interactions (different effects from each combination of components). The analyses will be performed under a Bayesian framework. There are no established methods for sample size calculations for component network meta-analysis.

For the secondary outcomes assessed via an online or telephone survey, data screening strategies were employed during survey development to minimize incomplete or inaccurate responses. These strategies included the use of mandatory fields (i.e., participants were unable to leave a survey item blank, but could select ‘prefer not to say’), minimizing the inclusion of open responses and reducing the survey length. Best practice recommendations for data screening will also be employed following data collection, including visually inspecting data to identify data entry errors or implausible values for each variable, and calculating distributional characteristics of items to assist in identifying outliers or extreme values (41).

### Trial governance

The trial will be overseen by a Steering Group, comprised of representatives from each LHD, including: Aboriginal Health Promotion Managers; program developers, implementation scientists, trialists and research dietitians from the University of Newcastle. Roles and responsibilities will be documented in a Terms of Reference for the Group. LHDs will be responsible for the selection of implementation strategies for their jurisdiction, and execution of some of the strategies to schools. The University of Newcastle will be responsible for facilitating trial workshops, ethics, data collection, monitoring and quality assurance, data management and analysis. A Community of Practice, established in the pilot, (26) will also be employed to support the interpretation of trial results and pooled analyses, exchange tacit knowledge and experience and identify opportunities for improvement.

## Discussion

This protocol provides a comprehensive description of a novel research design, employing individual level participant (i.e., ‘school’) data component meta-analysis, to help generate evidence that can better inform approaches to support the adoption and implementation of health promotion interventions at scale. The study will provide rigorous evidence of the effects of a variety of implementation strategies, employed in different contexts on the adoption of the SWAP IT school lunchbox program.

Evidence generated from this research will help address an important constraint of the current literature, with systematic review evidence identifying few rigorous trials that have tested strategies to implement health promotion interventions at scale. The strategies tested within this study have been developed following a systematic co-design approach with implementation researchers, LHD health promotion staff and other stakeholders. In addition to considering the evidence-base (i.e., barriers and enablers to adoption of school-based programs, and the effectiveness of implementation strategies), this process included working with LHD health promotion staff to consider the human, technical and financial resources available in LHDs responsible for strategy delivery. Applying this type of systematic approach to scale up has been recommended by implementation and scale-up experts to help address a common pitfall of scale-up research, which is the diminishment in effect of interventions with proven efficacy when delivered at scale (42, 43).

The currently limited evidence base has resulted in a failure to provide guidance on the crucial issue of context, with strategies that effectively improve implementation in one context potentially ineffective or inappropriate to deliver in another (22). Through partnering with 13 NSW LHDs (including three from the pilot) to conduct this research, schools from all sectors and located within the majority (86%) of the state of NSW will be represented. These LHDs encompass socioeconomically and geographically diverse regions, ensuring the contexts in which these strategies are tested are diverse and representative of the broader setting (31). In order to further address the issue of context, future research should potentially consider identifying and addressing other contextual, sectoral and political factors that may be influential in maximizing school adoption of the SWAP IT program. For example, the World Health Organization’s Health Promoting Schools Framework recommends employing a comprehensive approach, encompassing education (e.g., learning and curriculum), environmental (e.g., culture and policies) and partnership (e.g., families, health professionals and educators, teachers and community) components, to enhance the effectiveness of health promotion programs (44). Employing individual-level participant data component meta-analysis within this research provides an opportunity to gather robust evidence on the types of strategies that are effective in improving implementation of SWAP IT, and in what context. Additionally, it addresses a noticeable constraint in the current literature, that is, the substantial heterogeneity in trial design and measures employed in the few studies that have tested strategies to implement health promotion programs at scale. IPD meta-analysis is considered the gold standard for combining data from randomized trials and has several advantages over other analytical approaches (45–47). These advantages include the increase in statistical power compared to aggregate data meta-analysis, the ability to standardize the analysis across studies to ensure consistency in outcome measures, and enhancing the ability to effectively explore heterogeneity in participant characteristics (i.e., schools and LHDs) and treatment effects (i.e., implementation strategies) (45–47). This type of analysis has been frequently employed to synthesize the effects of health behavior interventions (48–50). For example, the Transforming Obesity Prevention for CHILDren (TOPCHILD) Collaboration uses IPD meta-analysis to assess the effectiveness of obesity-prevention interventions on child weight outcomes, and also assess differential effects by individual- and trial-level characteristics (50).

The use of objective and validated measures of data collection to assess study outcomes is a considerable strength of the study. For example, the objective measure of school adoption of SWAP IT, automatically captured upon school registration for the program, will provide high-quality and accurate assessment of the trial primary outcome. The use of validated measures (38) within the survey to assess school acceptability of the employed implementation strategies will provide reliable insight into the types of strategies that could potentially be employed within future interventions to support the implementation of health promotion programs. The use of such measures has been recommended by leading implementation researchers, who have developed definitions and validated measures of implementation outcomes (including adoption and acceptability) to improve the consistency in how outcomes are assessed within the implementation field and enable the comparison of strategies across studies (38, 51). These definitions and measures have been incorporated within other school-based interventions to assess implementation outcomes (52, 53). Despite the strengths outlined above, a number of limitations should be considered. While employing a Master Protocol trial design is innovative and shows promise as a method to transform traditional approaches to evaluating strategies to improve implementation of health promotion programs, there is also limited research to guide the conduct of such trials in school-based interventions. As such, the utility of this type of trial design in school-based interventions is still largely unknown. Indeed, the study will provide valuable learnings of this design as a model of evidence generation more broadly. Additionally, although the analysis will include schools from 13 of the 15 LHDs, these schools are solely located within one state of Australia. As such, generalizability of the findings beyond this region may be limited.

## Ethics statement

The studies involving humans were approved by Hunter New England (2019/ETH12353); University of Newcastle (09/07/26/4.04); NSW of Department of Education (2018247); and the Maitland-Newcastle, Sydney, Wollongong, Bathurst, Parramatta, Wagga Wagga and Canberra-Goulburn Catholic Dioceses. The studies were conducted in accordance with the local legislation and institutional requirements. The participants provided their written informed consent to participate in this study.

## Author contributions

CB: Conceptualization, Investigation, Methodology, Project administration, Writing – original draft, Writing – review & editing. JJ: Investigation, Methodology. Project administration, Writing – original draft, Writing – review & editing. LW: Conceptualization, Investigation, Methodology, Project administration, Writing – original draft, Writing – review & editing. KR: Conceptualization, Investigation, Methodology, Project administration, Writing – original draft, Writing – review & editing. AS: Formal analysis, Methodology, Writing – original draft, Writing – review & editing. JN: Investigation, Methodology, Project administration, Writing – original draft, Writing – review & editing. PB: Investigation, Methodology, Project administration, Writing – original draft, Writing – review & editing. MM: Investigation, Methodology, Project administration, Writing – original draft, Writing – review & editing. CP: Investigation, Methodology, Project administration, Writing – original draft, Writing – review & editing. LM: Writing – review & editing, Investigation, Methodology, Project administration, Writing – original draft. JM: Investigation, Methodology, Project administration, Writing – original draft, Writing – review & editing. JP: Investigation, Methodology, Project administration, Writing – original draft, Writing – review & editing. SM: Investigation, Methodology, Project administration, Writing – original draft, Writing – review & editing. SD: Investigation, Methodology, Project administration, Writing – original draft, Writing – review & editing. CV: Investigation, Methodology, Project administration, Writing – original draft, Writing – review & editing. ND: Investigation, Methodology, Project administration, Writing – original draft, Writing – review & editing. KM: Investigation, Methodology, Project administration, Writing – original draft, Writing – review & editing. RS: Conceptualization, Investigation, Methodology, Project administration, Writing – original draft, Writing – review & editing.
