# Supplementary figures and images for: A collaborative network trial to evaluate the effectiveness of implementation strategies to maximize adoption of a school-based healthy lunchbox program: a study protocol
Source: Front Public Health. 2024 Mar 27;12:1367017. doi: 10.3389/fpubh.2024.1367017 (PMC11004312; doi:10.3389/fpubh.2024.1367017)

**SUPPLEMENTARY FILE 2: SPIRIT Schedule**


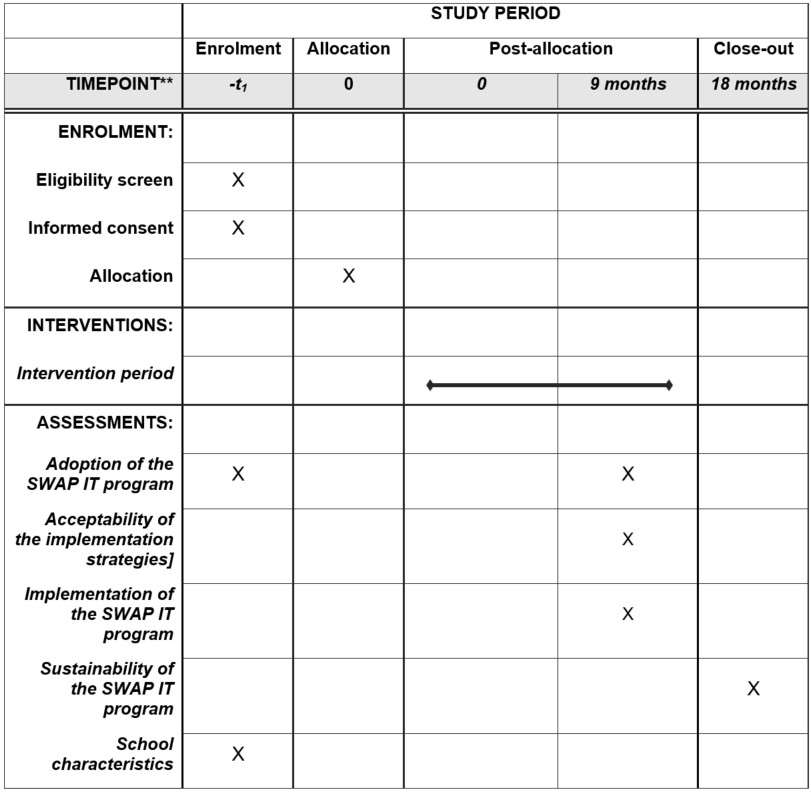

Supplement: Supplementary file 2 [file Table_2.docx]
